# Metatranscriptomic Analysis of the Chicken Gut Resistome Response to In-Feed Antibiotics and Natural Feed Additives

**DOI:** 10.3389/fmicb.2022.833790

**Published:** 2022-04-14

**Authors:** Raju Koorakula, Matteo Schiavinato, Mahdi Ghanbari, Gertrude Wegl, Nikolaus Grabner, Andreas Koestelbauer, Viviana Klose, Juliane C. Dohm, Konrad J. Domig

**Affiliations:** ^1^Department of Food Science and Technology, Institute of Food Science, University of Natural Resources and Life Sciences, Vienna, Austria; ^2^Competence Centre for Feed and Food Quality, Safety and Innovation (FFoQSI), Tulln, Austria; ^3^Department of Biotechnology, Institute of Computational Biology, University of Natural Resources and Life Sciences, Vienna, Austria; ^4^DSM – BIOMIN Research Center, Tulln, Austria

**Keywords:** metatranscriptomics, gut microbiome, resistome, chicken, antibiotic resistance genes, phytogenic feed additives

## Abstract

The emergence of resistance against common antibiotics in the gut microbiota is a major issue for both human and livestock health. This highlights the need for understanding the impact of such application on the reservoir of antibiotic resistance genes in poultry gut and devising means to circumvent the potential resistome expansion. Phytogenic feed additives (PFAs) are potential natural alternative to antibiotic to improve animal health and performance, supposedly *via* positively affecting the gut microbial ecosystem, but there is little systematic information available. In this time-course study, we applied a shotgun meta-transcriptomics approach to investigate the impact of a PFA product as well as the commonly used antibiotic, zinc bacitracin either at AGP concentration or therapeutic concentration on the gut microbiome and resistome of broiler chickens raised for 35 days. Over the course of the trial, PFA treatments increased the abundance of *Firmicutes* such as *Lactobacillus* and resulted in a lower abundance of *Escherichia*, while the latter group increased significantly in the feces of chickens that received either AGP or AB doses of bacitracin. Tetracycline resistance and aminoglycoside resistance were the predominant antibiotic resistance gene (ARG) classes found, regardless of the treatment. PFA application resulted in a decrease in abundance of ARGs compared to those in the control group and other antibiotic treatment groups. In summary, the findings from this study demonstrate the potential of phytogenic feed additives could be an alternative to antibiotics in poultry farming, with the added benefit of counteracting antimicrobial resistance development.

## Introduction

Antibiotic resistance is one of the most serious global threats to human health, so immediate action is needed to tackle the current situation and reduce its spread (Sabino et al., 2019). Antibiotics have been used for decades in livestock, both at subtherapeutic (low-dose) levels to promote growth and at therapeutic (high-dose) levels against diseases (Castanon, 2007; Looft et al., 2014; Van Boeckel et al., 2015; Mehdi et al., 2018; Sun et al., 2018; Ghanbari et al., 2019). In poultry farming, low-dose antibiotics (antibiotic growth promoters, “AGPs”) have been used for many years to increase nutrient uptake efficiency, for growth performance, to maintain bird health (Butaye et al., 2003; Danzeisen et al., 2011; Page and Gautier, 2012; Costa et al., 2017; Kumar et al., 2018) and to prevent enteric diseases (Butaye et al., 2003; Miles et al., 2006; Wei et al., 2013). However, recent studies suggest that this practice can contribute to the emergence of antimicrobial-resistant bacteria (ARBs), accelerating the antibiotic resistance problem in animal and human pathogens (Butaye et al., 2003; Diarra and Malouin, 2014; Costa et al., 2017; Ghanbari et al., 2019). Moreover, therapeutic dose administration of antibiotics may be subinhibitory for some host-associated bacteria, enhancing the selection for antibiotic resistance genes and their horizontal transfer (von Wintersdorff et al., 2016). Hence, poultry farmers are facing the challenge of finding alternatives to antibiotic growth promoters (Inglis et al., 2005; Doyle and Erickson, 2012; Lawley et al., 2013; Looft et al., 2014). However, zinc-bacitracin is still one of the most commonly used AGP in poultry farming (Sarmah et al., 2006; Crisol-Martínez et al., 2017) and is usually included in feed at non-therapeutic doses of <55 mg/kg body weight (KBW) to improve growth performance and reduce early mortality (Diarra and Malouin, 2014). Treatment with a higher dosage of 55–110 mg/kg body weight (KBW) is used to prevent and treat necrotic enteritis caused by *Clostridium perfringens*, which has a high mortality rate and is one of the most economically significant gut diseases in broiler chickens (Butaye et al., 2003). Bacitracin, a polypeptide antibiotic obtained from *Bacillus licheniformis*, is a mixture of high molecular weight cyclic peptides (bacitracins A, B, C and other minor compounds) that have antibacterial action against gram-positive microorganisms by interfering with cell wall development and the synthesis of peptidoglycan (Phillips, 1999; Marshall and Levy, 2011; Díaz Carrasco et al., 2018).

Due to their ability to mimic the bioactive properties of antibiotics, phytogenic feed additives (PFAs) are a possible alternative to AGPs (Murugesan et al., 2015; Salaheen et al., 2017). Phytogenics are plant-derived natural substances (herbs, spices, oils or extracts) that contain sensory and flavoring compounds. They are added to animal diets to improve animal health and feed acceptance (Wang J. et al., 2021) and have been linked to improved gut health, better nutrient digestibility, and increased growth performance (Murugesan et al., 2015; Kaschubek et al., 2018; Bampidis et al., 2019; Wang J. et al., 2021). There is, however, paucity of research on the effect of the PFAs application on gut microbiome of livestock species such as broiler chickens. By applying a shotgun meta-transcriptomics approach, in this study we systematically studied the impact of a PFA product as well as the commonly used antibiotic, zinc bacitracin at two different concentration (either as low dose AGP or high dose treatment) on the gut microbiome and resistome of broiler chickens. The findings of this study have important implications for broiler production and public health, since such analysis provided a deeper insight on the effect of the AGP and therapeutic doses of antibiotics on the antibiotic resistome. In addition, the analysis demonstrated the potential of using the natural alternative to antibiotics in poultry farming on mitigating antimicrobial resistance development.

## Materials and Methods

### Animals and Experimental Design

The animal trial was carried out at the Center of Animal Nutrition (Tulln, Austria) under a protocol approved by the office of the Lower Austrian Region Government, Group of Agriculture and Forestry, Department of Agricultural Law (approval code LF1-TVG-57/005-2018) according to relevant guidelines and regulations. Two hundred forty-old male broiler chickens (Ross 308) were randomly assigned to 24 pens with 10 birds per pen, and then the pens were randomly assigned to one of six treatment groups, each with four replicating pens. The chickens were fed a standard broiler diet for 35 days *ad libitum*. Treatments were started after 3 days of adaptation, and the six groups received the following: (1) a standard diet of basal chicken feed [Control (CON), (2) supplementation with a phytogenic feed additive (Digestarom^®^ DC Power, Biomin Holding GmbH, Austria; 150 mg/kg feed) throughout the trial (PFA), (3) supplementation with zinc-bacitracin (ALBAC, Huvepharma, Belgium) as an antibiotic growth promotor at 20 mg/kg throughout the trial (AGP), (4) supplementation of both the PFA and AGP groups throughout the trial (AGP + PFA), (5) a basal diet with an antibiotic intervention with zinc-bacitracin (200 mg/kg)] administered from Day 15 to Day 21 (AB), and (6) phytogenic supplementation as in the PFA group with the additional antibiotic intervention of the AB group.

### Feces Sampling

On Day 3, before the switch to supplemented feed, one bird per pen was euthanized by asphyxiation, and following dissection, chicken digesta samples from the distal part of the colon (herein referred to as feces) were collected. Due to the low amount of digesta in such young animals, the four replicates of each treatment were pooled. As all birds still received the same diet at Day 3, the pooled samples from chickens were considered six replicates of the same condition and were used as Day 3 samples for all the treatments. On later sample Days 14, 21, and 35, two birds per pen were euthanized for sampling, and their combined homogenized digesta were counted as one sample. Samples were snap-frozen on dry ice and stored at −80°C for later processing (Peimbert and Alcaraz, 2016; Song et al., 2016). RNA was extracted from samples within 1 week. A total of 78 samples were collected (6 treatments × 3 sampling points × 4 replicates + 6 Day 3 pool samples).

### RNA Extraction and Quantification

Extraction of total RNA was performed using the RNeasy Power Microbiome kit, QIAGEN GmbH, Hilden, Germany) following the manufacturer’s instructions with some minor modifications: the input material was reduced to 150 mg fecal biomass, and RNA was finally eluted in 80 μL of RNAse-free water. After RNA isolation, RNA was quantified using the Qubit RNA XR Assay Kit on a Qubit 4.0 fluorometer (Invitrogen™, United States), while the RIN was determined using the Bioanalyzer RNA 6000 Nano assay (Agilent) on the Bioanalyzer 21000 system (Agilent, Santa Clara, CA, United States). Extracted RNA was stored at −80°C until further use.

### Library Construction and RNA Sequencing

All samples of chickens were sent for RNA sequencing to the Vienna Biocenter Core Facilities (VBCF-NGS, Vienna, Austria). Single-end sequencing cDNA libraries were prepared from the extracted RNA from chicken samples using standard Illumina library preparation with the NEBNext® Ultra™ RNA Library Prep Kit (Illumina Inc., San Diego, CA, United States), and rRNA was removed with the Ribo-Zero Magnetic Gold (Epidemiology) Kit (Epicentre Biotechnologies), followed by sequencing on an Illumina NovaSeq 6000 S1 FlowCell 100 cycle platform using high-output chemistry (1 × 100 bp) according to the manufacturer’s protocol.

### Filtering of Raw Reads

Raw sequencing reads from each of the 78 samples were quality-filtered with Trimmomatic (Bolger et al., 2014) (ILLUMINACLIP:TruSeq2-SE.fa:2:30:10, LEADING:20, TRAILING:20, SLIDINGWINDOW: 4:26, MINLEN:50). Bowtie2.4.2 (Langmead and Salzberg, 2012) was used to map the reads against the chicken reference genome (*Gallus gallus* release 90, downloaded from Ensembl) and the Phix174 bacteriophage genome to filter out host and contaminating reads. Ribosomal RNA was removed with SortMeRNA (Kopylova et al., 2012) based on the 16S, 18S, 23S, 28S, 83 5S, and 5.8S rRNA databases.

### Resistome Annotation

Filtered reads were then assigned to antibiotic resistance genes (ARGs) based on sequence identity to known resistance genes contained in the MegaRES2 database with the AMR + + pipeline (Doster et al., 2019) using the “with RGI_Kraken” workflow adjusted to run with single-end reads. Each read was assigned uniquely to the ARG with which it had the highest sequence identity.

Assigned reads were also screened for the presence or absence of single nucleotide polymorphisms (SNPs) that could remove the resistance power of a certain ARG. For this, we adapted the workflow of this pipeline to work with single-end short reads and to properly perform SNP confirmation on them. In fact, certain ARGs are present in different alleles, and only some of them produce antibiotic resistance. Hence, AMR + + also performs a SNP confirmation step whereby it removes those reads that do not show any SNPs associated with antibiotic resistance, despite having been assigned to an ARG (Doster et al., 2019). A table containing the number of reads assigned to each ARG was obtained for each sample. The counts relative to each gene in this table refer to non-deduplicated reads that passed the SNP confirmation step (performed within AMR + +). The results of each of the 78 samples were merged in a single table with a custom Python script.

### Gene Expression

A comprehensive table of non-deduplicated, SNP-confirmed counts was used to detect differentially expressed ARGs at each timepoint (D3, D14, D21, D35) between each feeding program. To extract differentially expressed ARGs, DESeq2 was used (Love et al., 2014). On D14, D21 and D35, four replicates per treatment were included. ARGs with low average read counts (<10) across all samples were filtered out since they are known to produce background noise in false discovery rate estimations (Stephens, 2016). The statistical significance of each differentially expressed gene was assessed using the “results()” function of DESeq2, calculating two-tailed *p*-values (altHypothesis = “greaterAbs”) and using the Benjamini-Hochberg correction (pAdjustMethod = “BH”). Only significant (*p* < 0.05) differentially expressed ARGs were considered. Log2FoldChange values were shrunken using the “lfcShrink” DESeq2 function, as suggested by the DESeq2 guidelines, using the “ashr” method (Love et al., 2014).

DESeq2 was also used to produce a table of fragments per kilobase per million mapped reads (FPKM). The FPKM values were then converted to transcripts per million (TPM) values using a conversion formula (Pachter, 2011). A TPM table was then used to extract ARGs that were uniquely expressed in certain feeding programs and timepoints. TPM values were averaged among replicates of the same condition (i.e., feeding program + timepoint). A custom Python script based on the pandas and numpy modules was used to extract the unique ARGs, considering as expressed only those genes with an average TPM ≥ 1.

### Taxonomy

Quality-trimmed reads were assigned to taxa using Kraken2 (Wood et al., 2019) (–minimum-hit-groups 2 –confidence 0.0). Taxa counts were then normalized and combined at the genus level with Bracken (Lu et al., 2017) (−r 100 −l G). The Bracken database was built with “bracken-build” using a read length of 100 and a k-mer size of 50 (−k 50 −l 100). Read counts per taxon were used as raw counts to assess differential taxa presence between each condition (i.e., timepoint + treatment) and the untreated D3 samples. The same was also done when comparing the treatments PFA (Digestarom®), AGP (low-dose bacitracin), AGP + PFA (low-dose bacitracin + digestarom), AB (high-dose bacitracin) and AB + PFA (high-dose bacitracin + digestarom) against the CON (control) at each timepoint. The same pipeline used for differential gene expression was used (see above), using genera as entries instead of genes. The same statistical approach was used to determine significance.

### Relative Abundance of Antibiotic Resistance Genes and Taxa

Antibiotic resistance genes were annotated with their Type, Class, Group and Mechanism from the MEGARES v2 database, each representing a different level of their functional characterization. TPM expression values, which are normalized by sequencing depth and gene length, were scaled to a (0,1) interval to represent their relative abundance in each sample. Scaling was performed by dividing each TPM value by the sum of the TPM values of the corresponding sample. Relative abundances from replicates of the same condition (i.e., timepoint + group) were averaged. The procedure was performed at the class, group and mechanism annotation levels. Given the large number of classes, groups and mechanisms, the top 10 classes (or groups or mechanisms) were represented independently, and the remaining classes were grouped together under the “Other” label. The top 10 entries were selected by averaging the relative abundance of each entry across all conditions and sorting the means decreasingly. All these operations were performed with a custom python (v3.6.4) script using the pandas (v1.0.1) and plotnine (v0.6.0) modules.

The relative abundance of taxa was determined similarly. Given the large number of unclassified reads, only classified reads were retained to improve further data visualization. Taxonomic abundance was assessed using the normalized counts produced by bracken at the genus and phylum levels. Unrelated counts belonging to the “Arthropoda,” “Chordata” or “Mollusca” phyla were discarded. The relative abundances were then rescaled to a (0,1) interval.

### Alpha and Beta Diversity

Principal coordinate analysis (PCoA), richness, diversity, dissimilarity and non-metric multidimensional scaling (NMDS) ordinations were calculated for ARG expression and for taxa (in both cases using read counts). The operations were performed in a custom python (v3.6.4) code using the following modules: pandas (v1.0.1), numpy (v1.18.1), de_toolkit (v0.9.12), sklearn (v0.23.0), and skbio (v0.5.6). Principal coordinates (skbio.stats.ordination.pcoa, method = “eigh”) were computed from a matrix containing Euclidean distances between samples (scipy.spatial.distance.pdist, metric = “braycurtis”). Richness (“observed_otus”) and diversity (“shannon”) were computed in both subsets for each condition (i.e., timepoint + treatment), using the “alpha_diversity” function of the skbio python module. Significance levels (*p* < 0.05) in comparisons between richness and diversity values among treatments were assessed in an R script with a Mann–Whitney *U* test using the R function wilcox.test. Dissimilarity (“braycurtis”) was calculated with the “beta_diversity” function of skbio. Multidimensional scaling was performed with the “manifold.MDS” function of the sklearn python module (n_components = 2, dissimilarity = “precomputed,” metric = False) (alpha = 0.05). Significant differences (*p* < 0.05) between NMDS ordinations were calculated pairwise between different groups of samples using a permanova test performed within a python script using the permanova function contained in the skbio.stats.distance module. Plots were generated with the plotnine python module and the ggplot2 R library (Wickham, 2016).

## Results

### Sequencing Data Overview

The sequencing generated approximately 1.3 billion Illumina single-end transcript reads, ranging from 8 to 18 million reads per sample (100 bp read length). On average, 82% of the raw reads passed the quality control. In detail, approx. eight percent of the reads were removed due to low quality (Phred score < 33), approx. six percent of the reads were classified as rRNA, and ∼ 4% of reads were classified as host reads (*Gallus gallus*) or PhiX bacteriophage reads. Filtered reads were then assigned to antibiotic resistance genes (ARGs) based on sequence identity to known resistance genes contained in the MegaRES2 database. Assigned reads were also screened for the presence or absence of single-nucleotide polymorphisms (SNPs) that could remove the resistance power of a certain ARG (see section “Materials and Methods”).

### Gut Resistome Diversity and Composition

**FIGURE 1:**
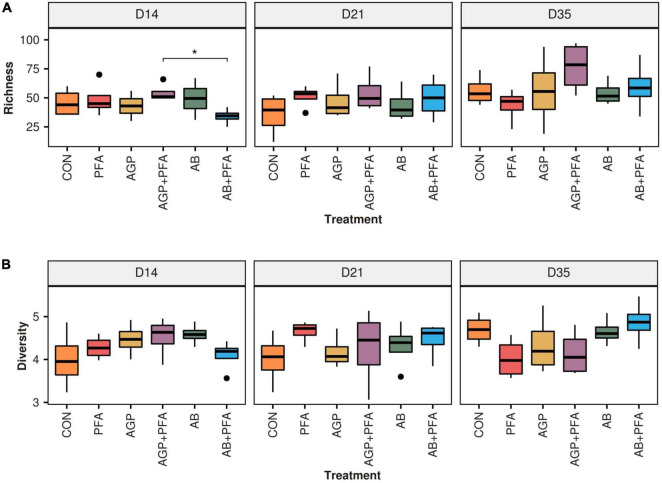
**(A)** Richness and **(B)** diversity (Shannon) of ARGs across feeding treatments (CON, PFA, AGP, AGP + PFA, AB, AB + PFA) and sampling points (D14, D21, D35). Bars in the boxplot represent interquartile ranges (25th to 75th percentile). The horizontal black line represents the median. Whiskers show intervals going from –1.5 to + 1.5 of the interquartile range. Dots indicate values falling outside of the interquartile range. Significance was tested with a Mann–Whitney *U* test (“*” = 0.05). Colors indicate different treatments.

The reads were assigned to 506 different ARGs out of the 7,868 ones contained in the MEGARes2 database. Considering all chicken samples together, 271 of the detected ARGs belonged to type “Drugs,” 120 to type “Metals,” 75 to type “Multicompound” and 40 to type “Biocides.” For the sake of this analysis, we focused only on ARGs assigned to the “Drugs” type, which encompass 11 classes of resistance and are involved in 25 mechanisms. From the raw read counts, we obtained normalized expression values in terms of transcripts per million (TPM) (Supplementary Data File 1). We measured alpha diversity metrics within each treatment in terms of ARG richness (i.e., number of ARGs represented) and diversity (Shannon index, i.e., evenness of the expression levels among ARGs). The results are summarized in Supplementary Data File 1 - ARG_RICHNESS, ARG_DIVERSITY and Figure 1.

The overall size (i.e., richness) of the resistome was not affected by treatments when compared to the control (CON). However, AGP + PFA-treated chicken samples exhibited a significantly higher richness (*p* < 0.05) than the AB + PFA combination at Day 15 (Figure 1A). In general, ARG richness had a heterogeneous distribution across replicates, which likely affected the significance testing Overall, the richness ranged from 25 ARGs in AB + PFA at Day 14 to 76.5 ARGs in samples of chickens treated with AGP + PFA on day 35. In terms of Shannon diversity, there was no statistically significant difference among the treatments (Figure 1B), although, numerical difference in diversity was observed in samples of chickens treated with AB + PFA at Day 35 (mean: 4.8 ± 0.50), with respect to the samples of chickens treated with CON at Day 14 (mean: 4.0 ± 0.68).

**FIGURE 2:**
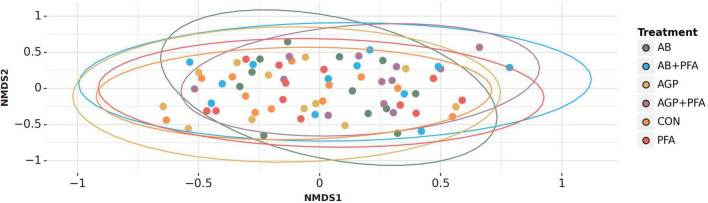
NMDS ordination based on Bray-Curtis dissimilarity metric represents bacterial compositional differences between the treatments.

We performed non-metric multidimensional scaling (NMDS) ordinations based on Bray–Curtis dissimilarities at gene level to assess the differences in composition between the treatments and timepoints, which did not show any specific clustering of the analyzed factors (Figure 2, Supplementary Data File 1 and Supplementary Data File 3 – see Supplementary Figures 1–3 for further details).

**FIGURE 3:**
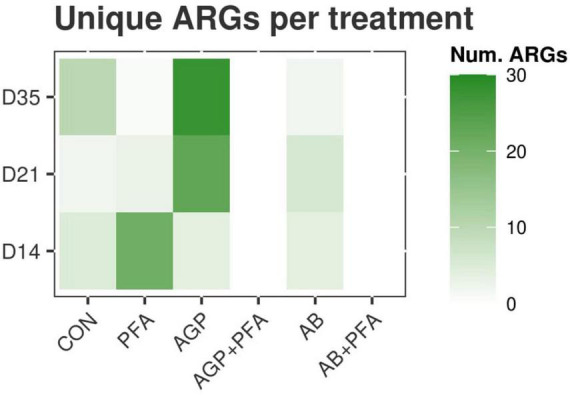
Heatmap representing the number of unique ARGs found in each treatment that were not found in any other treatment at any given timepoint.

We then set out to determine whether certain ARGs were uniquely expressed in a certain treatment when compared to other treatments at any given timepoint. TPM values determined in previous experiments were used. The results are summarized in Figure 3 and in Supplementary Data File 1 – ARG_UNIQUE_CLASS. At Day 14, samples of chickens treated with PFA showed 21 unique ARGs that were not found in the other treatment groups. Of these 21 were 14 unique ARGs belonging mostly to aminoglycosides. Samples derived from chickens that were fed AGP or AB harbored four unique ARGs, three of which belong to the tetracycline class. The control samples showed five unique ARGs, most of which belong to the tetracycline class (Figure 3).

At Day 21, 23 unique ARGs was found in the samples from chickens treated with AGP, and these unique ARGs belong to the tetracycline, macrolide-lincosamide-streptogramin (MLS), and multidrug classes. Samples from chickens treated only with AB contained six unique ARGs belonging to the MLS and aminoglycoside classes. The other treatments showed three or fewer unique ARGs (Figure 3).

At Day 35, a very high number of unique ARGs (28) was found for samples of chickens treated with bacitracin given as AGP. These ARGs belong mostly to the aminoglycosides, sulfonamides or MLS classes. CON samples showed 10 unique ARGs. Other treatments showed two or fewer unique ARGs (Figure 3). Samples of chickens treated with the combinations AGP + PFA or AB + PFA contained no unique ARGs (0) at Days 14, 21, and 35 (Figure 3; Supplementary Data File 1 – ARG_UNIQUE_CLASS).

**FIGURE 4:**
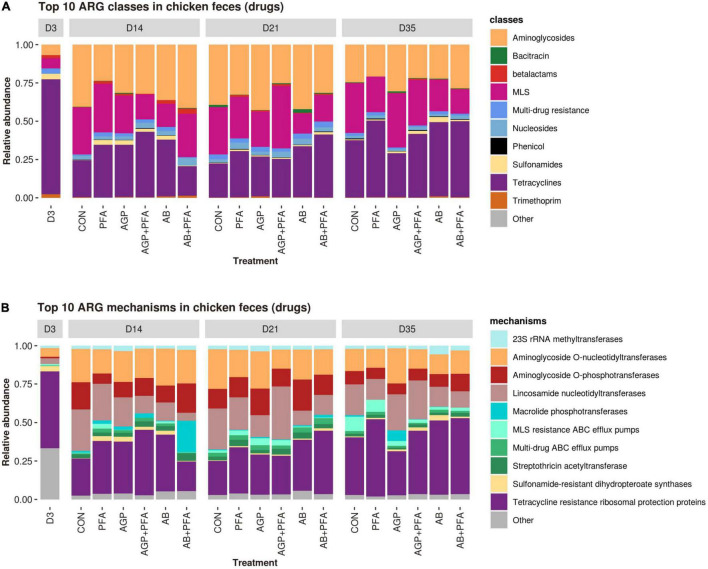
Relative transcript abundances of the top 10 ARG classes **(A)** and mechanisms **(B)** in chicken fecal samples from all feeding treatments (CON, PFA, AGP, AGP + PFA, AB, AB + PFA) and sampling points (D3, D14, D21, D35). The ARG classes and mechanisms with a relative abundance of <1% of the total reads were grouped into “Other”. To facilitate comparisons, the D3 timepoint was represented as a single feeding treatment alongside the other six because all samples at D3 could be considered replicates.

The ARGs detected in the read dataset were then analyzed in terms of relative transcript expression based on their expression level calculated in TPM (transcripts per million). Overall, tetracycline resistance was the predominant class to which reads aligned (71 ARGs), followed by aminoglycoside and MLS resistance (64 ARGs and 52 ARGs, respectively) (Supplementary Data File 1 – ARG_RELATIVE_ABUNDANCE_CLASS and Figure 4A). In the tetracycline class, the predominant mechanism of resistance observed was resistance through ribosomal protection proteins (RPPs), represented by 64 ARGs. Other mechanisms were aminoglycoside O-nucleotidyltransferases, aminoglycoside O-phosphotransferases, lincosamide nucleotidyltransferases and MLS resistance AB efflux pumps, all of which were found at high levels (Supplementary Data File 1 –ARG_RELATIVE_ABUNDANCE_MECHANISM and Figure 4B). Overall, only a few differences were observed between the treatments and sampling points in terms of relative transcript abundance of ARG classes and mechanisms. Bacitracin resistance genes were present throughout the entire period in almost all the samples from chickens at very low abundance (<0.5% for the majority of samples). Bacitracin supplementation given as antibiotic intervention at a high dose, however, revealed a relative abundance of 2% on Day 21. In fact, regardless of the bacitracin application, all the treatments harbored a diverse range of ARGs at all sampling points (Figure 4).

**FIGURE 5:**
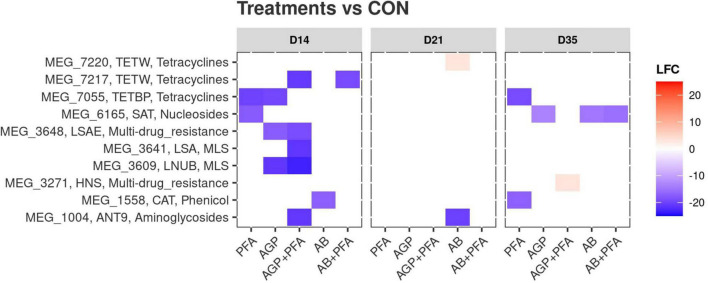
Differential expression of antibiotic resistance genes after administration of treatments and compared against the control (CON). *X*-axis: treatments by timepoint; “CON” represents the control sample. *Y*-axis: gene IDs as per the MEGARes2 database, together with their group and their class. The color reflects the detected log2foldChange (LFC) value. Only significantly (*p* < 0.05) differentially expressed genes are shown.

We then determined differentially expressed ARGs between each treatment and the control sample from the same sample day (CON) at any given timepoint (Supplementary Data File 1 – ARG_DEGS and Figure 5).

All the treatments were first compared against the control (CON) at given any timepoint. We identified a total of 10 differentially expressed ARGs in all the treatments (Figure 5 and Supplementary Data File 1). These 10 ARGs produced 20 instances of differential expression, as certain genes (MEG1004_ANT9 – aminoglycosides and MEG1558_CAT-Phenicol) were found to be differentially expressed in multiple comparisons. Considering that each comparison was independent from the others, we treated them as 20 independent differentially expressed genes. Of these 20 instances of differential expression, two included increases in differential abundance (Figure 5, red tiles). These included MEG 3271, a gene of interest belonging to the Resistance-Nodulation-Division (RND) multidrug resistance class [Histone-like Nucleoid Structuring (HNS) proteins]. This gene showed a significant increase in gene expression [Log2foldchange (LFC) ∼ 3.5, *p* < 0.05] after AGP + PFA administration at Day 35. The second gene with increased expression was MEG 7220 (LFC ∼ 3.2, *p* < 0.05), belonging to the tetracycline resistance class, after AB treatment at Day 21 (Figure 5 and Supplementary Data File 1 – ARG_DEGS). The remaining 18 instances of differential expression included decreases in abundance. AGP + PFA-treated samples showed a higher number of differentially expressed ARGs (*p* < 0.05) (for more details refer Supplementary Data File 1 – ARG_DEGS and Figure 5) at Day 14 than at Day 21 and Day 35 in all treatments. Interestingly, MEG_7055-TETB (tetracycline) and MEG_1588-CAT (phenicol) ARGs had significantly decreased expression after PFA administration at Day 35. In detail, MEG 7055-TETB showed an LFC ∼−19.3, while MEG 1588-CAT showed an LFC ∼−17 (both genes with *p* < 0.05).

All treatments were then compared to each other over timepoints. A richer collection of instances of differential expression (168) that corresponded to 20 different ARGs was found. For example, MEG_3271 (RND multidrug resistance class) was increased in abundance when comparing chicken treated with AGP + PFA against the PFA treatment alone (LFC ∼ 3.6, *p* < 0.05, Supplementary Data File 1 – ARG_DEGS). We did not find any changes in transcript abundance at the ARG class or gene level that directly corresponded to bacitracin administration at low or high doses. Instead, we observed that many of the ARGs belonging to the tetracycline class were significantly increased in abundance and that many of the aminoglycoside classes were significantly decreased in abundance in response to high-dose bacitracin administration at Day 21 (Supplementary Data File 1 – ARG_DEGS).

### Microbiome Diversity and Gut Composition

We then assigned the quality-trimmed RNA-Seq reads to their most likely taxonomic origin. With the read counts per taxon, we assessed the relative abundance, richness, and diversity of taxa in each sample (Supplementary Data File 2).

**FIGURE 6:**
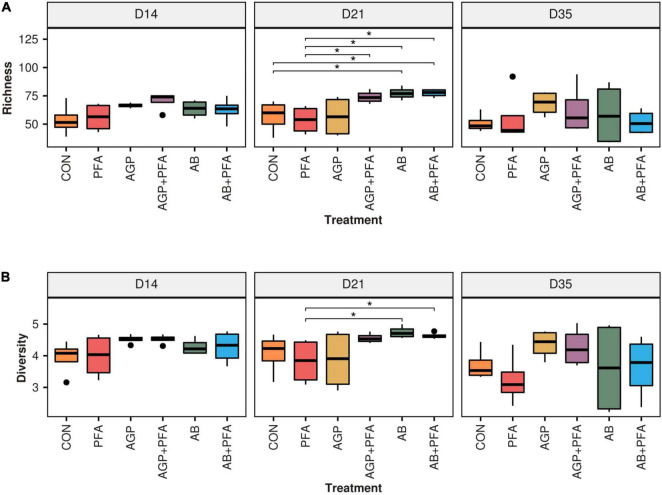
**(A)** Richness and **(B)** diversity (Shannon) of taxa across treatments and over sampling points. Bars in the boxplot represent interquartile ranges (25th to 75th percentile). The horizontal black line represents the median. Whiskers show intervals going from –1.5 to + 1.5 of the interquartile range. Dots indicate values falling outside of the interquartile range. Significance was tested with a Mann–Whitney *U* test (“*” = 0.05). Colors indicate different treatments.

The results showed that the total number of identified genera (richness) and the evenness of their abundance (Shannon diversity) were different (only at numerical level) in samples of chickens that received antibiotics alone or in combination with phytogenics (AGP, AGP + PFA, AB, AB + PFA) than in those treated with PFA only or the control (CON) (Figure 6A). In fact, chickens treated with AB or AB + PFA showed a significantly higher ARG richness in the feces than CON (control) and PFA treated chickens at Day 21 (*p* < 0.05; Figure 6A). Additionally, chickens treated with AGP + PFA showed a significantly higher ARG richness in the feces than those that received PFA at Day 21 (*p* < 0.05; Figure 6A).

When looking at the diversity (Figure 6B), samples derived from chickens treated with CON and PFA showed numerically different median value (Shannon) than those from bacitracin-treated chickens (AGP, AGP + PFA, AB, AB + PFA), this difference was not statistically significant though. However, high-dose bacitracin-treated chickens (AB and AB + PFA) had a significantly higher diversity in their feces than those treated with PFA at Day 21.

Further assessment of the microbiome composition and diversity across the treatments and timepoints after NMDS ordinations based on the Bray–Curtis dissimilarity displayed no clear separation between treatments and timepoints (Figure 2, Supplementary Data File 2 and Supplementary Data File 3 – see Supplementary Figures 4, 5). However, a permanova test revealed that six comparisons between control and non-control samples were significantly different (*p* < 0.05) at Day 21: AGP + PFA vs. CON; AGP + PFA vs. PFA; AB vs. CON; AB vs. PFA; AB + PFA vs. CON; AB + PFA vs. PFA. This indicates that there were significant compositional differences of the microbes in the feces of bacitracin-treated animals (AGP, AGP + PFA, AB, AB + PFA), control (CON), and PFA-treated animals at Day 21 (Supplementary Data File 2).

**FIGURE 7:**
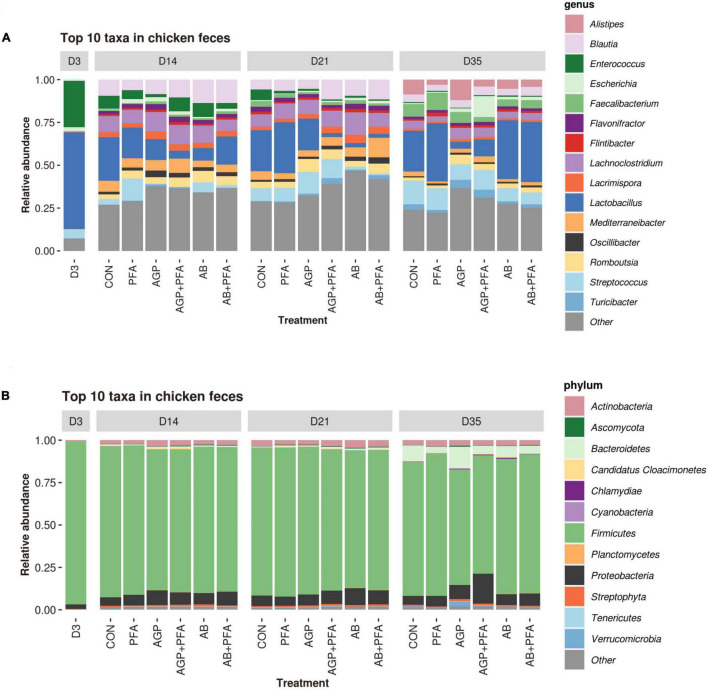
Relative abundance of the most abundant taxa (≥1% of the total reads) in each treatment at each time point at the genus **(A)** and phylum **(B)** levels; taxa representing < 1% of the total reads were grouped together under the label “Other”.

Taxonomic profiling was carried out to determine whether the temporal changes in ARG profiles were related to the changes in the fecal microbial communities in response to PFA, AGP, and AB administration. Figure 7 shows the distribution of the most prevalent phyla and genera in the fecal samples over the feeding trial (Supplementary Data File 2 – TAXA _RELATIVE_ABUNDANCE_PHYLUM and TAXA_RELATIVE_ABUNDANCE_GENUS). Firmicutes was by far the most predominant phylum, followed by *Proteobacteria, Actinobacteria*, and *Bacteroidetes*. At Day 35, *Bacteroidetes* and *Proteobacteria* were found at high relative abundance in broiler chickens treated with AGP and AGP + PFA, respectively.

At the genus level, samples from chickens treated with PFA (Digestarom^®^) exhibited a high relative abundance of *Lactobacillus* (probiotic), and AGP-treated animals also displayed an increased abundance of *Escherichia* throughout the feeding trial (from Day 14 to 35). At Day 21, AGP + PFA (low-dose bacitracin + digestarom), AB (high-dose bacitracin) and AB + PFA (high-dose bacitracin + digestarom)-treated chickens showed a steep decrease in the abundance of the *Lactobacillus* genus, and AB and AB + PFA samples also showed a low abundance of *Streptococcus*. At Day 35, the genus *Lactobacillus* was at low relative abundance, while the *Escherichia* genus displayed high abundance in the samples of chickens treated with AGP and AB + PFA (Figure 7A). Interestingly, the abundance of the *Lactobacillus* and *Streptococcus* genera in samples of chickens derived from the AB or AB + PFA treatments recovered at Day 35, 2 weeks after antibiotic (high-dose bacitracin) withdrawal (Figure 7A).

**FIGURE 8:**
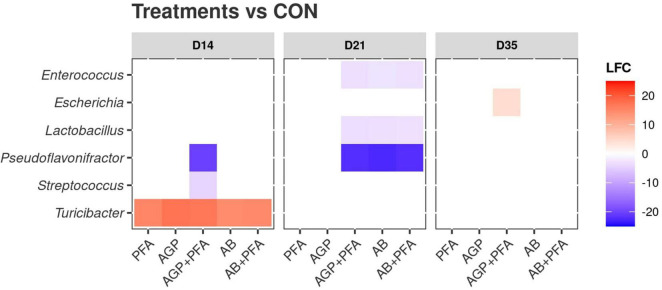
Differential abundance of bacterial genera compared against the control (CON). *X*-axis: treatments by timepoint; “CON” represents the control sample. *Y*-axis: genera. The color reflects the detected log2foldChange (LFC) value. Only significant (*p* < 0.05) differences in taxa abundance are shown.

We then calculated the differential abundance of each detected taxon in each treatment against the control (CON; Figure 8). The results are summarized in Supplementary Data File 2 – TAXA_DEGS. The analysis at the genus level showed 85 instances of significant differential abundance across all comparisons (e.g., PFA vs. AGP, etc.), which all referred to six genera (*Lactobacillus, Enterococcus, Escherichia, Streptococcus, Turicibacter*, and *Pseudoflavonifractor*). The majority of these instances regarded *Lactobacillus* and *Pseudoflavonifractor* (24 and 27 instances, respectively). At Day 14, all treated samples showed a significantly higher abundance of *Turicibacter* than that of the control (CON). Moreover, chickens treated with AGP + PFA also exhibited a decrease in the abundance of the genera *Pseudoflavonifractor* and *Streptococcus*. At Day 21, chickens treated with AGP + PFA, AB and AB + PFA showed a general decrease in the *Enterococcus, Lactobacillus* and *Pseudoflavonifractor* genera when compared to the control. At Day 35, an increase in *Escherichia* was found in samples of chickens treated with AGP + PFA compared to CON chickens (LFC > 10, *p* < 0.05).

When comparing treatments against each other (and not against CON), at Day 21, the *Lactobacillus* genus was significantly (*p* < 0.05) more abundant in chickens treated with PFA (Digestarom^®^) than in those treated with AGP + PFA, AB or AB + PFA (Supplementary Data File 2 – TAXA_DEGS).

## Discussion

Antimicrobial resistance gene spread in livestock has reached alarming levels in most parts of the world and has now been recognized as a significant emerging threat to global public health and food security (Hawkey, 2008; Van Boeckel et al., 2017). Resistance patterns among bacteria have traditionally been studied using culture on media selecting for resistant bacteria. However, when we move away from the most well-studied pathogens, the vast majority of microorganisms cannot be cultured, at least not by standard methods (Browne et al., 2016; Lau et al., 2016). The recent advances in culturomics (Lagier et al., 2018; Nowrotek et al., 2019) and next-generation sequencing techniques (Crofts et al., 2017; Lanza et al., 2018), have made it possible to investigate the resistome in specific bacteria or in bacterial populations at an unprecedented depth. Shotgun whole-metagenome sequencing (WMS) is a reliable tool that can provide a comprehensive and high-resolution analysis of the microbiome and resistome (Schmieder and Edwards, 2011; Nesme et al., 2014; Ghanbari et al., 2019; Ma et al., 2021), and it has been applied to quantify the abundance of many resistance genes in parallel in poultry (Wang et al., 2017; Kumar et al., 2020), cattle (Thomas et al., 2017), pig (Ghanbari et al., 2019; Mencía-Ares et al., 2020) and the human gut (Feng et al., 2018). Other metagenomic studies have found multiple ARGs in chicken gut as well (Tong et al., 2017; Xiong et al., 2018; Eckstrom and Barlow, 2019; Wang Y. et al., 2021; De Cesare et al., 2022). However, WMS provides only limited information regarding ARG activity or the overall functional profile of the active microbial community. Shotgun whole-metatranscriptome sequencing (WMTS) is therefore needed at the population level to determine whether the predicted ARGs are partially or fully expressed. For example, one study that used combined metagenomics and metatranscriptomics on human, pig, and chicken gut resistomes by Wang et al. (2020) observed that ARG transcripts have different abundances when compared to their ARG gene abundance. Interestingly, a study by Franzosa et al. (2014) showed that across the subjects, metatranscriptomic functional profiles were more individualized than metagenomic data. In the current study, we focused on the expression of antibiotic resistance genes in microbial communities of the chicken gut receiving AGP, therapeutic agents (ABs), and/or a phytogenic feed additive (PFA) using WMTS.

Our diversity results revealed that in all treatments, including the control, a diverse range of antibiotic resistance genes was expressed, even in the absence of antibiotic pressure and regardless of the antibiotic choice. The common ARG classes, encoding tetracycline and aminoglycoside resistance, as well as MLS resistance, were the most prevalent ARGs in all treatments, including the control (Figure 4). The expressed ARGs found in this study were similar to what was found in chicken feces in previous metagenomics studies by Xiong et al. (2018) and Wang et al. (2020) and in chicken cecum by Juricova et al. (2021). This result supports the theory that ARGs are not spread randomly in different environments (Xiong et al., 2018) but rather that there exists a high background level of the gut resistome in chickens because antibiotics have been used for five decades in poultry, both at the subtherapeutic and therapeutic levels (Tong et al., 2017).

We found that bacitracin administration as an antibiotic growth promoter (AGP) or therapeutic agent (AB) and a phytogenic feed additive (PFA) did not show any significant impact on the alpha diversity of the resistome. We speculate that this is due to general differences in the level of expression of ARGs, which would have only marginally affected common diversity indices or could have been a result of limited statistical power due to the low sample size (*n* = 4; Figure 1). However, on Day 21, bacitracin administration at a high dose (AB) resulted in a detectable increase in the relative expression of the bacitracin ARG class, which was even higher than the high background resistance, although the gut resistome diversity had mostly recovered after 2 weeks of antibiotic withdrawal. A study from Gupta et al. (Gupta et al., 2021) found an increase in the relative abundance of bacitracin resistance in hens given bacitracin, which is consistent with our findings.

Interestingly, we found that AGP-treated chicken samples showed a continuous increase in the number of unique ARGs over the course of the feeding trial. In contrast, PFA-treated chickens showed a decrease in the number of unique ARG transcripts over time. Additionally, antibiotic combination with phytogenics (AGP + PFA; AB + PFA) showed no unique ARG transcripts over the feeding trial. These findings are supported by those from previous studies and indicate that administration of AGP (low-dose antibiotics) in feed causes an accumulation of ARGs in complex ecosystems (Butaye et al., 2003; You and Silbergeld, 2014; Salaheen et al., 2017; Gupta et al., 2021). Overall, the most expressed ARG classes in our study (aminoglycosides, MLS, tetracyclines) are known to be prevalent in the chicken gut resistome (Li B. et al., 2015; Wang et al., 2020; Wang Y. et al., 2021). However, the results from another study by Gupta et al. (2021) indicated that multidrug and beta-lactam ARGs were most abundant. In our study we did not observe high expression of these ARG classes. The differential abundance analysis revealed increased expression of the MEG_3271 gene in samples from chickens treated with AGP + PFA (Figure 5). This gene belongs to the multidrug resistance class of the RND multidrug resistance efflux pump mechanism. This change may be attributed to the detected significant increase in the transcript abundance of *Escherichia* (Figure 7) after AGP + PFA administration at Day 35. In fact, in a previous study, the *Escherichia* genus was found to be the most common host for multidrug resistance ARGs (Li B. et al., 2015; Xiong et al., 2018; Afridi et al., 2020). While interesting, we note that this may very well be also due to the fact that Escherichia coli is a popular model organism. Interestingly, at Day 21 and Day 35, we found that samples of chickens treated with PFA showed more ARGs decreased in expression when compared to the control group and to other antibiotic treatment groups (Supplementary Data File 1 – ARG_DEGS). At Day 35, the transcript abundance of the tetracycline-TETBP and phenicol-CAT ARG class genes decreased significantly in PFA-treated animals compared to those in CON- or AB + PFA- and AB-treated animals, respectively.

Taxonomically, *Firmicutes* was the most prevalent phylum, accounting for more than 90% of the bacterial population in all treatments throughout the feeding trial (Figure 7). Other abundant phyla were *Proteobacteria, Actinobacteria*, and *Bacteroidetes.* Similar findings were reported in other studies (Videnska et al., 2013; Becker et al., 2014). Moreover, *Bacteroidetes* and *Proteobacteria* showed increased relative abundance in the feces of bacitracin-treated chickens. Compositional shifts in the bacterial communities were mainly observed at Day 21 (after 7 days of antibiotic administration) in all the treated chicken samples, including the control. It may be possible that these changes are the result of AGP or AB administration, as found by previous studies that showed that AGP or AB administration alters the composition of chicken gut microbiota (Díaz Carrasco et al., 2018; Kumar et al., 2018; Proctor and Phillips, 2019). The microbial diversity analysis revealed that a high-dose bacitracin treatment increased both the bacterial community richness and diversity at Day 21. A significant increase in richness was also observed in samples of chickens treated with AGP + PFA (Figure 6). These results align with those obtained by Crisol-Martínez et al. (2017) and suggest that a marked reduction in predominant taxa such as *Lactobacillus* in chickens treated with bacitracin correlates with increased bacterial community richness and diversity. On the other hand, these results differ from those observed in the microbial community of the chicken cecum when treated with bacitracin, where an increase of *Lactobacillus* was detected (Díaz Carrasco et al., 2018). The antimicrobial activity of phytogenic feed additives (PFAs) has been studied using metagenomics (Dorman and Deans, 2000; Mitsch et al., 2004), but only a few studies have examined how they could aid the proliferation of beneficial bacteria (Jamroz et al., 2005; Mountzouris et al., 2011). The results of our study revealed that animals treated with PFA showed an increased abundance of active bacteria associated with probiotic properties such as those belonging to the *Lactobacillus* genus over the course of the trial (Figure 7A). Interestingly, the abundance of the genus *Escherichia* increased significantly in the feces of chickens receiving AB + PFA at Day 35. In a previous study (Murugesan et al., 2015), it was shown that PFA (i.e., Digestarom^®^) promoted the development of beneficial gut microbiota with higher numbers of *Lactobacillus* than those in low-dose bacitracin (AGP)-treated chickens. Multiple studies have documented that the *Lactobacillus* genus is an outstanding probiotic, preventing enteric diseases by selectively excluding pathogens from adhering and promoting poultry health by stimulating the immune system (Lutful Kabir, 2009; Mountzouris et al., 2011; Díaz Carrasco et al., 2018).

## Conclusion

Over the course of the trial, the phytogenic feed additive (Digestarom^®^) increased the abundance of genera from the *Firmicutes* phylum such as *Lactobacillus* and *Faecalibacterium.* It also resulted in lower abundance of *Escherichia*, whereas low-dose bacitracin treatment administered together with digestarom increased the abundance of the *Escherichia* genus. We speculate that this could be connected to the observed increase in the abundance of multidrug resistance genes such as efflux pumps because *Escherichia* is known to have a high prevalence of multidrug resistance ARGs (Li H. L. et al., 2015; Xiong et al., 2018). In addition, *Alistipes* was significantly increased in the feces of the chickens which received either AGP (low-dose bacitracin) and AB (high-dose bacitracin) or AB + PFA (high-dose bacitracin + digestarom). Of note, administration of the phytogenic feed additive (PFA) significantly decreased the gene expression of ARGs in the feces of the chickens compared to those in the control group and other antibiotic treatment groups.

To the best of our knowledge, this is the first chicken gut meta-transcriptomic study that focused on the impact of different diets containing a phytogenic feed additive and bacitracin at different dosages and combinations. Our study highlighted the trends in resistome gene expression and the active microbiota composition that resulted from each treatment, showing that treatment with PFA (Digestarom^®^) could be a good candidate alternative to low-dose bacitracin treatments (AGPs) in poultry, which are banned in the EU.

## Data Availability Statement

The datasets presented in this study can be found in online repositories. The names of the repository/repositories and accession number(s) can be found in the article/Supplementary Material.

## Ethics Statement

The animal study was reviewed and approved by the animal trial was carried out at the Center of Animal Nutrition (Tulln, Austria) under a protocol approved by the office of the Lower Austrian Region Government, Group of Agriculture and Forestry, Department of Agricultural Law (approval code LF1-TVG-57/005-2018) according to European Guidelines for the Care and Use of Animals for Research Purposes (European Council, 2010). Written informed consent was obtained from the owners for the participation of their animals in this study.

## Author Contributions

MG and KD contributed to the conception and design of the project. RK performed the experiments. MS and RK performed the data analysis, interpreted the data, and wrote the manuscript. MS conducted the statistical analysis. NG and AK supported the experiments. MG, GW, JD, and KD supervised the development of the work and helped in data interpretation. GW, AK, VK, JD, MG, and KD reviewed the manuscript and provided critical suggestion and comments. All authors have read and approved the final manuscript.

## Conflict of Interest

MG, GW, NG, AK, and VK are employed by DSM Animal Nutrition & Health, which provided support in the form of salaries for the authors but did not have the main role in the experimental design, data collection and analysis, decision to publish, or preparation of the manuscript. DSM Animal Nutrition & Health is involved in natural feed additive development and research in natural alternatives to in-feed medication in livestock production. The remaining authors declare that the research was conducted in the absence of any commercial or financial relationships that could be construed as a potential conflict of interest.

## Publisher’s Note

All claims expressed in this article are solely those of the authors and do not necessarily represent those of their affiliated organizations, or those of the publisher, the editors and the reviewers. Any product that may be evaluated in this article, or claim that may be made by its manufacturer, is not guaranteed or endorsed by the publisher.
