# Supplementary material for: Metatranscriptomic Analysis of the Chicken Gut Resistome Response to In-Feed Antibiotics and Natural Feed Additives
Source: Front Microbiol. 2022 Apr 14;13:833790. doi: 10.3389/fmicb.2022.833790 (PMC9048739; doi:10.3389/fmicb.2022.833790)
Supplement: Supplementary file 3 [file Data_Sheet_3.docx]

**Supplementary Data**


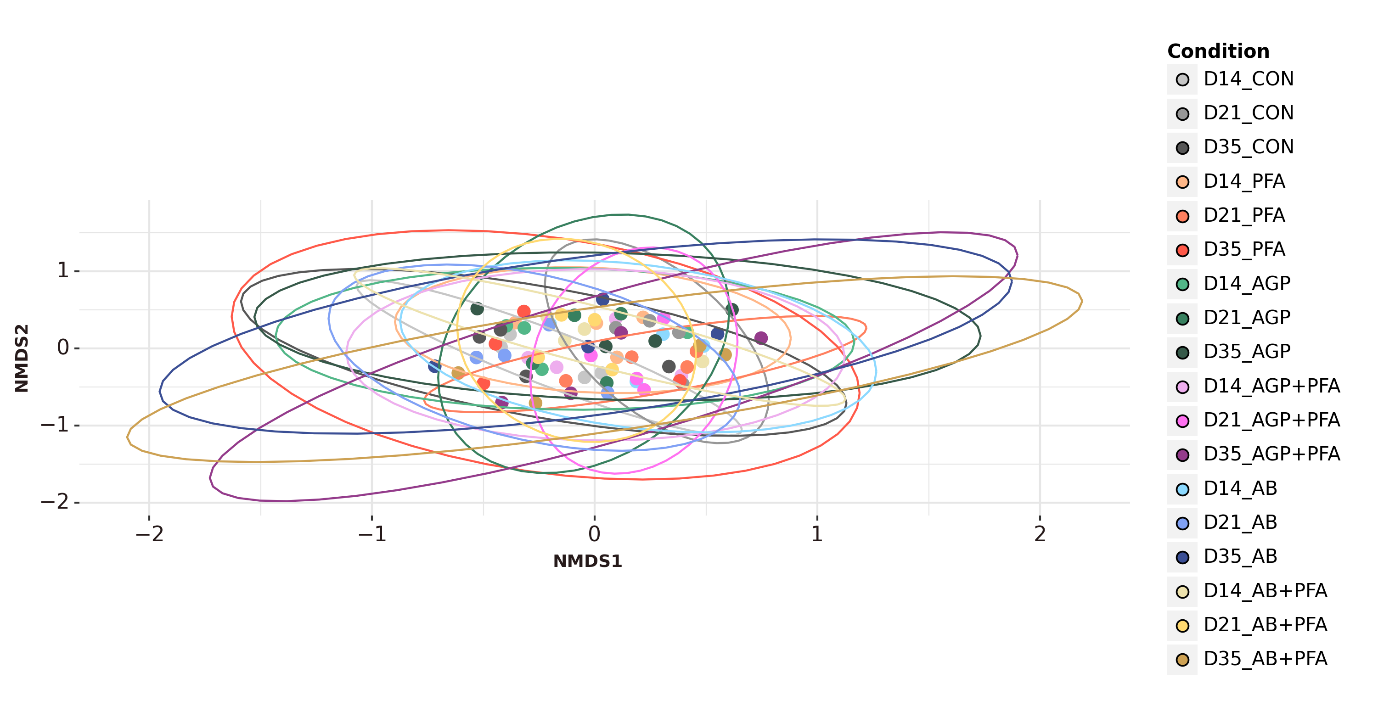


**Figure - S1** NMDS ordination based on Bray-Curtis dissimilarity metric represents ARGs compositional differences in treatments over sampling points


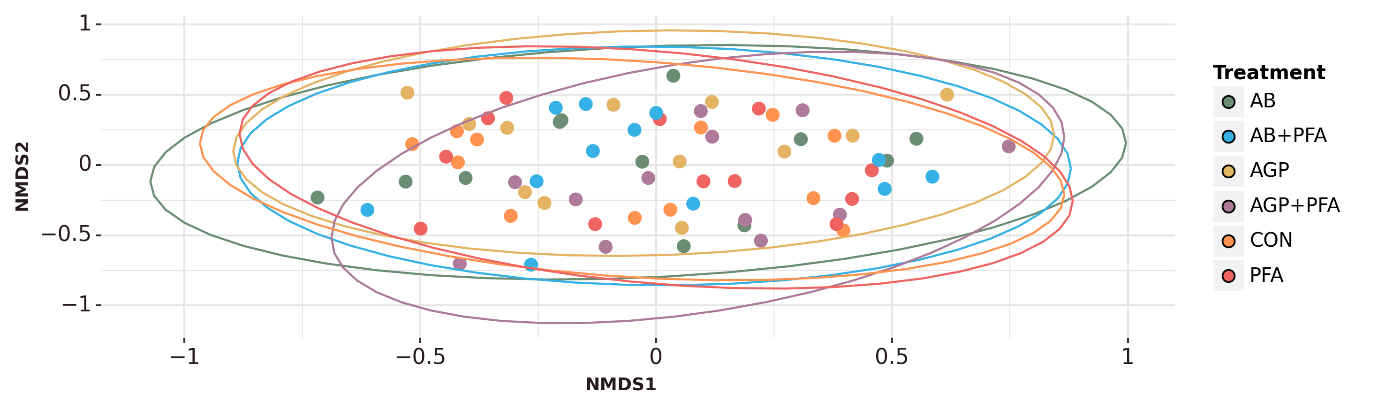


**Figure S2** - NMDS ordinations based on Bray–Curtis dissimilarity metric showing the changes in ARGs compositions between the treatments.


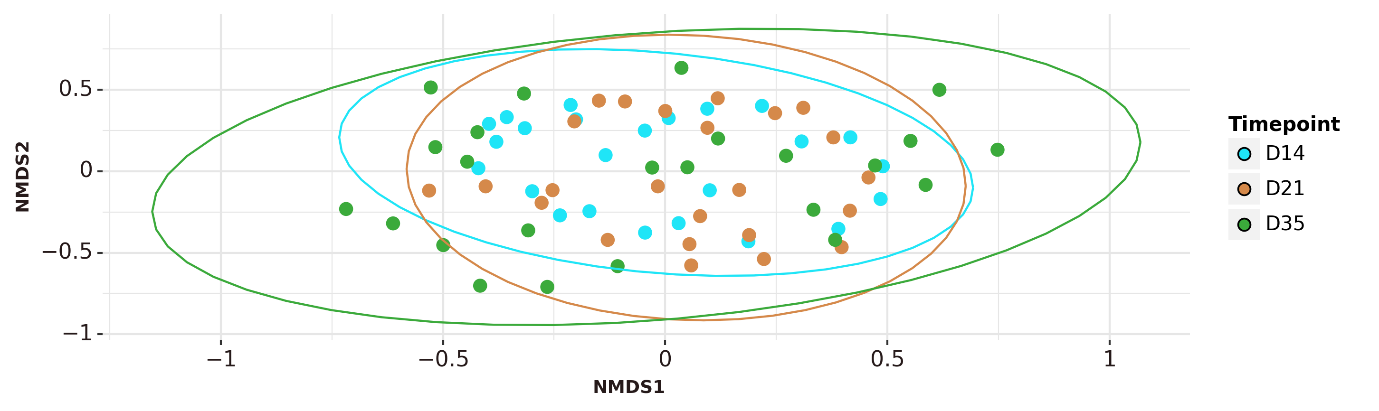


**Figure S3** - NMDS ordination based on Bray-Curtis dissimilarity metric represents ARGs compositional differences between the timepoints.


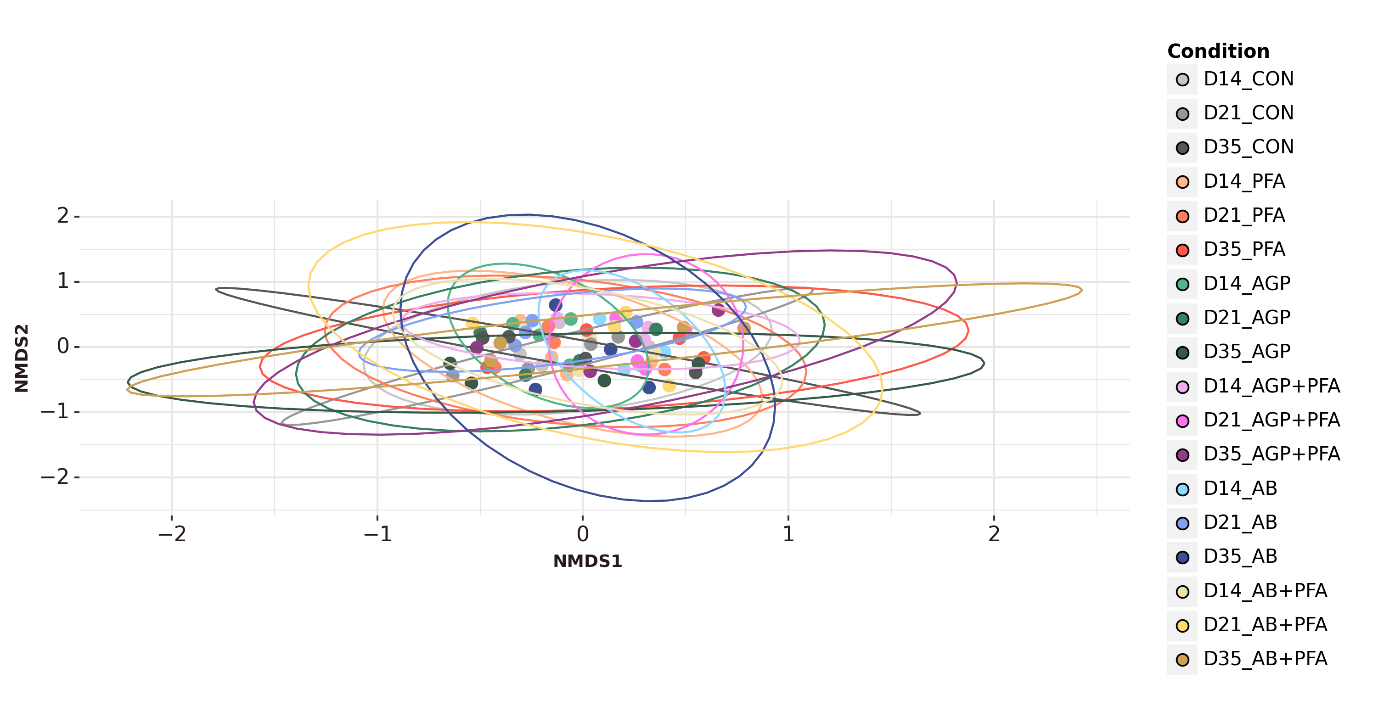


**Figure S4** - NMDS ordination based on Bray-Curtis dissimilarity metric represents bacterial compositional differences in treatments over time points


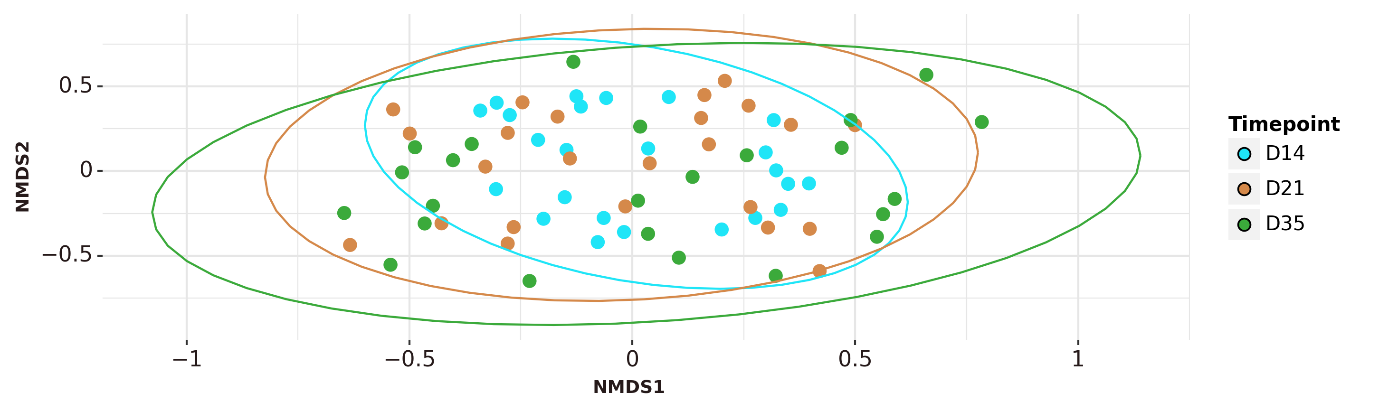


**Figure S5** - NMDS ordination based on Bray-Curtis dissimilarity metric represents bacterial compositional differences between the timepoints.
